# Comparison of Hemodynamic Visualization in Cerebral Arteries: Can Magnetic Resonance Imaging Replace Computational Fluid Dynamics?

**DOI:** 10.3390/jpm11040253

**Published:** 2021-03-30

**Authors:** Minh Tri Ngo, Ui Yun Lee, Hojin Ha, Ning Jin, Gyung Ho Chung, Yeong Gon Kwak, Jinmu Jung, Hyo Sung Kwak

**Affiliations:** 1 Department of Radiology and Research Institute of Clinical Medicine of Jeonbuk National University, Biomedical Research Institute of Jeonbuk National University Hospital, Jeon-ju 54907, Korea; ngominu@gmail.com (M.T.N.); chunggh@jbnu.ac.kr (G.H.C.); kwakyg@jbnu.ac.kr (Y.G.K.); 2 Division of Mechanical Design Engineering, Jeonbuk National University, Jeon-ju 54896, Korea; euiyun93@naver.com; 3 Department of Mechanical and Biomedical Engineering, Kangwon National University, Chuncheon 24341, Korea; hojinha@kangwon.ac.kr; 4 Siemens Medical Solutions USA, Inc., Chicago, IL 60089, USA; ning.jin@siemens.com; 5 Hemorheology Research Institute, Jeonbuk National University, Jeon-ju 54896, Korea

**Keywords:** hemodynamics visualization, cerebral arteries, four-dimensional flow magnetic resonance imaging (4D flow MRI), signal intensity gradient from time-of-flight magnetic resonance angiography (TOF-MRA SIG), computational fluid dynamics (CFD)

## Abstract

A multimodality approach was applied using four-dimensional flow magnetic resonance imaging (4D flow MRI), time-of-flight magnetic resonance angiography (TOF-MRA) signal intensity gradient (SIG), and computational fluid dynamics (CFD) to investigate the 3D blood flow characteristics and wall shear stress (WSS) of the cerebral arteries. TOF-MRA and 4D flow MRI were performed on the major cerebral arteries in 16 healthy volunteers (mean age 34.7 ± 7.6 years). The flow rate measured with 4D flow MRI in the internal carotid artery, middle cerebral artery, and anterior cerebral artery were 3.8, 2.5, and 1.2 mL/s, respectively. The 3D blood flow pattern obtained through CFD and 4D flow MRI on the cerebral arteries showed reasonable consensus. CFD delivered much greater resolution than 4D flow MRI. TOF-MRA SIG and CFD WSS of the major cerebral arteries showed reasonable consensus with the locations where the WSS was relatively high. However, the visualizations were very different between TOF-MRA SIG and CFD WSS at the internal carotid artery bifurcations, the anterior cerebral arteries, and the anterior communicating arteries. 4D flow MRI, TOF-MRA SIG, and CFD are complementary methods that can provide additional insight into the hemodynamics of the human cerebral artery.

## 1. Introduction

Stroke is ranked as the world’s leading cause of death, with an annual mortality rate of approximately 5.5 million. This places stroke high on the 21st century public health agenda, highlighting its importance in health research [1]. Stroke is a disease affecting the arteries that lead to the brain and those within it [2]. Awareness of the hemodynamics of the human cerebral arteries is essential to better understand the mechanisms underlying disease initiation and progression.

In a specific vessel or lesion, a complete description of the hemodynamics requires a knowledge of the blood flow pattern. Abnormal patterns of blood flow, such as turbulent blood flow or transitional flow, may lead to disease progression [3]. Blood pressure, the natural stress acting against the vessel wall, has been commonly used as a vascular disease biomarker [4]. Currently, the wall shear stress (WSS) distribution in arterial flow is gaining attention due to emerging evidence that it is associated with atherosclerosis and vascular disease [5,6,7]. The effect of WSS on remodeling of arteries and its implication on vascular graft has become helpful in supporting clinical decision-making with regard to cardiovascular disease as well as designing new cardiovascular medical devices [5,8].

Nowadays, new radiological techniques have been introduced that assess the flow conditions in the circulatory system and that can demonstrate the pathophysiology of vascular disease [9]. Due to advances in the magnetic resonance (MR) technique and data processing capabilities, four-dimensional flow magnetic resonance imaging (4D flow MRI) has recently gained prominence. However, the restricted spatio-temporal resolution of 4D flow MRI sequences limits its clinical applications. Computational fluid dynamics (CFD) with image-based vasculature geometry extraction was used to obtain detailed knowledge of 3D fluid flow fields [10,11], but there was insufficient evidence that the results of the CFD simulation were physiologically true [12,13,14]. Han et al. [15] conducted a series of validation experiments on time-of-flight magnetic resonance angiography (TOF-MRA) signal intensity gradient (SIG) and showed that this approach is feasible to rapidly assess the arterial WSS. However, studies on the validation of this new method are rare.

This study presents an approach using 4D flow MRI, TOF-MRA SIG, and CFD as complementary methods to visualize the 3D blood flow pattern and arterial WSS of the cerebral arteries. The blood flow velocity of the major cerebral arteries was measured using 4D flow MRI. We used TOF-MRA data for the 3D CFD models and the flowrate obtained from the 4D flow MRI for the inlet boundary conditions in CFD simulation. Both CFD and 4D flow MRI were performed to visualize the 3D blood flow pattern. The maps of WSS described by CFD and TOF-MRA SIG were also compared.

## 2. Materials and Methods

### 2.1. Study Population

Sixteen healthy volunteers with no medical history of cardiovascular disease were included in this study. The mean age of the subjects was 34.7 ± 7.6 years. The protocol of this study was approved by our institutional review board (The Ethics Committee of Jeonbuk National University Medical School and Jeonbuk National University Hospital; JUH 2018-09-033).

### 2.2. Outline of the Workflow

**Figure 1:**
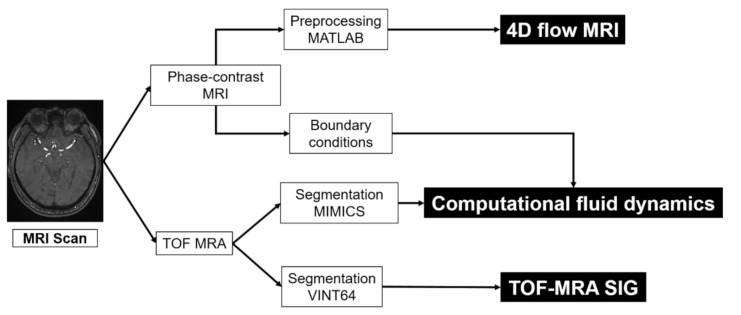
Flow chart of the multi-modality hemodynamic analysis of four-dimensional flow magnetic resonance imaging (4D flow MRI), computational fluid dynamics (CFD), and time-of-flight magnetic resonance angiography (TOF-MRA) signal intensity gradient (SIG).

The study method is illustrated in Figure 1. We used phase-contrast MRI (PC MRI) source data for the subject-specific 4D flow MRI study. The TOF-MRA source data were segmented and converted into 3D geometry for CFD simulations. The velocity obtained from the 4D flow MRI analysis was applied to CFD simulations as the boundary conditions. The TOF-MRA SIG was performed based on the TOF-MRA images. 4D flow MRI was used for measuring the blood flow velocity. Both CFD and 4D flow MRI were performed to visualize the 3D blood flow characteristics. Additionally, CFD and TOF-MRA SIG were used to represent the arterial WSS.

### 2.3. Imaging and Data Acquisition

A brain TOF-MRA scan was performed for each subject by using a Verio 3T MRI scanner (Siemens Healthcare, Erlangen, Germany). The scanning parameters used in this study were as follows: repetition time = 40.08 ms, echo time = 2.79 ms, field of view = 146–174 mm^2^, flip angle = 7°, slice thickness = 0.6 mm, matrix = 331–384, voxel = 0.84 × 0.84 × 0.03 mm. The acquisition time was 4–5 min.

After the TOF-MRA scan, 3D volumetric coverage of the cerebral arteries and 3-directional velocity encoding were performed in the PC MRI procedure. Prospective ECG gating was synchronized with data acquisition. The scanning parameters were as follows: repetition time = 18 ms, echo time = 3.11 ms, field of view = 151.6–151.6 mm^2^, flip angle = 20°, slice thickness = 3 mm, matrix = 155–256, spatial resolution = 1.2 × 1.2 × 1.2 mm.

The velocity encoding (Venc) of 100 cm/s was set. The total acquisition time was 15–20 min. The term 4D flow MRI refers to PC MRI, which includes all flow encoding resolved in all three dimensions of space with time (4D = 3D + time) during the cardiac cycle [16].

### 2.4. 4D Flow MRI Analysis

Using in-house software written in MATLAB (MathWorks, Natick, MA, USA), all 4D flow MRI data were preprocessed to remove Maxwell terms, eddy currents, and velocity aliasing, as described previously [17]. 3D blood flow characteristics and flowrate included in the resulting data were then analyzed by a 3D visualization and analysis software package (EnSight; CEI, Apex, NC, USA).

For the cerebral blood flow quantification, the 2D segmented planes were manually positioned at the anatomic landmarks of the major cerebral arteries within the anterior cerebral circulation: internal carotid artery (ICA, paraophthalmic segment), middle cerebral artery (MCA, middle M1 segment), and anterior cerebral artery (ACA, middle A1 segment). The mean (averaged over the vessel lumens that have been segmented) flow rate–time curves was calculated. For the 3D blood flow characteristics visualization, the 3D streamline was used, which displayed traces along a 3D velocity field at a particular point in time.

### 2.5. MRI Reconstruction and 3D Cerebral Arteries Model

Using Mimics software (version 21.0; Materialise NV, Leuven, Belgium), the obtained TOF-MRA DICOM images of sixteen cerebral arteries were reconstructed from 2D into 3D geometry using the thresholding method. Since the reconstruction method was crucial in this study, two experienced radiologists confirmed all the processes of segmentation. In this study, only cerebral arteries with anterior circulation were included using a crop mask tool. The edit mask in the 3D tool was employed to cut unnecessary branches. Before CFD simulation, all 3D cerebral artery models were smoothed.

### 2.6. Computational Fluid Dynamics Analysis

The geometry of the cerebral artery was exported in STL format file from Mimics and imported into COMSOL Multiphysics 5.2a software (COMSOL Inc., Burlington, MA, USA). By changing mesh element size parameters (maximum element size, minimum element size, maximum element growth rate, curvature factor, and resolution of narrow regions), the optimal number for mesh was found when there was no more variation in pressure and velocity. The meshes were formed with approximately 500,000 tetrahedral elements. The Reynolds number at peak systole ranged from 279 to 812 for the ICA. For the smallest vessel at ACA A1, the Reynolds number ranged from 30 to 540. The analyzed Reynolds number was confirmed to be similar to that previously reported [18]. The blood flow was assumed to be incompressible, laminar Newtonian fluid that followed the previously stated continuity equation [19]. At the inflows, the flow conditions derived from 4D flow MRI measurements at the ICAs were applied to the inlet boundary condition in the same arteries [20]. Three cardiac cycles were calculated and the second cycle was taken for the results. The traction-free boundary condition was applied at the outlets [21]. The arterial wall was assumed to be rigid with a no-slip condition [22]. Using the fully coupled method, the dependent variables such as pressure and velocity were calculated. For the numerical solving, iterative solver (generalized minimal residual algorithm (GMRES)) was used. The relative tolerance was set to 0.01 for the convergence of the solution.

3D streamline was used for the visualization of blood flow and WSS distributions were calculated for comparison with SIG [18]. To better display complex blood flow characteristics, all images in the figures are presented at peak systole.

### 2.7. Assessment of the TOF-MRA SIG

The workflow of TOF-MRA SIG analysis was described in previous publications [15,23]. The 2D source TOF-MRA images was imputed into the in-house-developed software (VINT64). Using this software, the 3D vascular geometry of the cerebral arteries was generated and segmented. The arterial segment from the supraclinoid ICA (ICA just after the branching site of the ophthalmic artery) to the MCA bifurcation and to the ACA A2 on the bilateral side was manually isolated. Finally, the SIG visualization of the segmented model was automatically performed. The time required for the TOF-MRA SIG analysis process was less than five minutes for each subject.

## 3. Results

### 3.1. Blood Flow Quantitative Analysis with 4D Flow MRI

The mean flow rates for the major arteries of the anterior cerebral circulation measured on 4D flow MRI data are summarized in Table 1. On an average, a flow rate measured with 4D flow MRI atthe ICA was 3.8 mL/s. The flow division at the bifurcation was 65.8% for MCA M1 (2.5 mL/s) and 31.6% for ACA A1 (1.2 mL/s).

### 3.2. 3D Blood Flow Patterns Derived Using 4D Flow MRI and CFD

**Figure 2:**
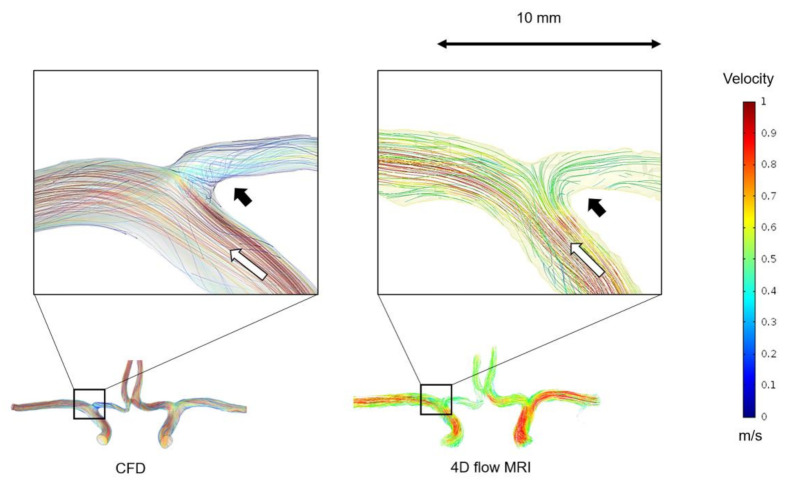
3D blood flow characteristics of the cerebral arteries obtained from CFD and 4D flow MRI. The major flow structures (white arrow) and the recirculation regions (black arrow) observed in the CFD coincided with those in 4D flow MRI. The same velocity scale bar with color was applied for all the images ranging from 0–1 m/s.

For all subjects, the results were obtained using 3D streamlines for a representative description of the characteristics of the systolic blood flow. For all models, the blood flow was well-visualized in the ICA, MCA, and ACA. Major flow structures and the recirculation regions observed in the CFD coincided with the 4D flow MRI, as shown in Figure 2. Generally, the blood flow patterns given by both methods showed reasonable consensus with the locations where the flow velocity was relatively low and where it was relatively high. However, similar to the reports of several previous studies [21,28,29,30,31], the maximum velocity magnitude of the blood flow tended to be lower in the 4D flow MRI models than in CFD simulations. The velocity fields from the CFD and 4D flow MRI showed large differences at ACA A2.

**Figure 3:**
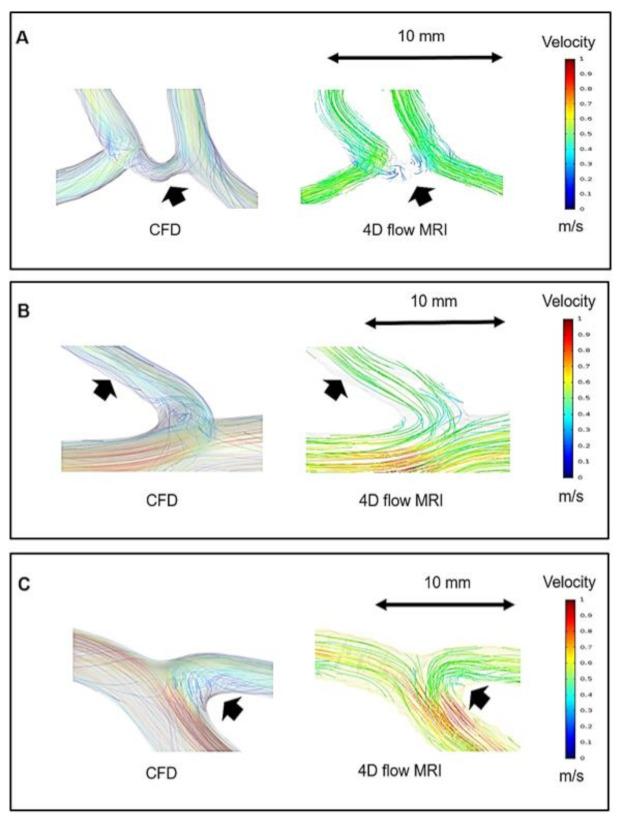
The differences in blood flow visualization between CFD and 4D flow MRI. (**A**) The blood flow in the anterior communicating artery (ACoA) is better visualized in CFD. (**B**) Low flows near the vessel wall are better captured by CFD. (**C**) Transitional flows at the ICA bifurcation visualized by CFD are of higher quality. The same velocity scale bar with color was applied for all the images ranging from 0–1 m/s.

Figure 3 identifies several differences between the two methods in the 3D blood flow characteristics: (1) The blood flow in the anterior communicating artery (ACoA) was better visualized in CFD than in 4D flow MRI; (2) low flows near the vessel wall were better captured by CFD; (3) recirculation region at the ICA bifurcation visualized by CFD was of higher quality compared to those by 4D flow MRI. These results reflect that CFD has the ability to deliver much higher resolution than 4D flow MRI.

### 3.3. Comparison of WSS Derived by CFD with TOF-MRA SIG

**Figure 4:**
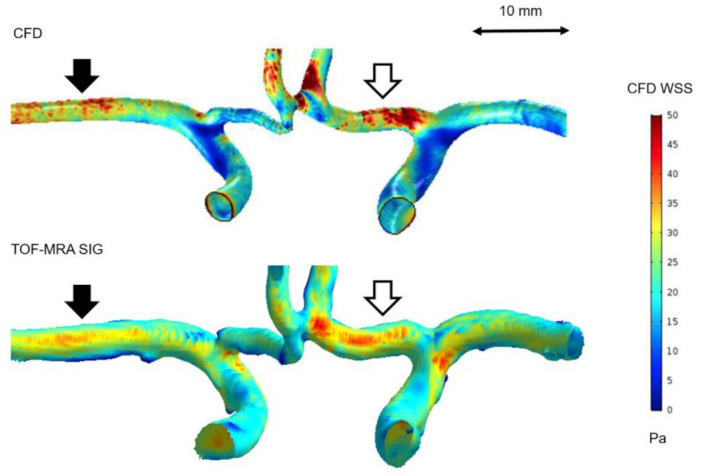
3D wall shear stress (WSS) mapping derived from CFD and TOF-MRA SIG. A consistency was seen in the regions of high WSS at the right middle cerebral artery (MCA) M1 (black arrows) and left anterior cerebral artery (ACA) A1 (white arrows).

**Figure 5:**
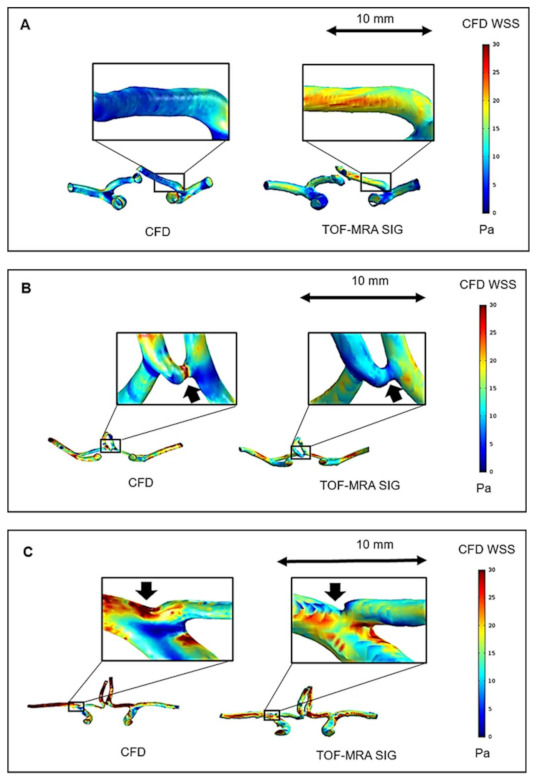
Comparison of CFD WSS with TOF-MRA SIG. The visualization was very different between the two techniques at the ACA (**A**), ACoA (**B**), and ICA bifurcation (**C**).

The TOF-MRA SIG for all subjects was visually compared with the WSS distribution derived by the subject-specific CFD simulation. Generally, we identified that the signal intensity of the arterial wall could provide specific hemodynamic information for each subject. However, although TOF-MRA SIG and CFD WSS of the major cerebral arteries showed fairly reasonable consensus with the locations where the arterial WSS was relatively high (Figure 4), the discrepancies between the two methods remained important in the detailed illustration of the WSS distribution. The visualization was very different between CFD WSS and TOF-MRA SIG for small vessels (i.e., ACA and ACoA) and the ICA bifurcation, as shown in Figure 5.

## 4. Discussion

In the present study, the use of 4D flow MRI was an efficient means of assessing the multidirectional blood flow of intracranial vessels. Compared with several reported data on healthy subjects (Table 1), the flow rates measured by employing the present approach were lower than published data of Wahlin et al. [24], Zarrinkoob et al. [25], and MacDonald et al. [26]. These findings, however, were mainly derived from manual delineation or semiautomatic thresholding of the vessel, and the partial volume effects were ignored. Typically, the critical zone theoretically influenced by partial volume effects is the peripheral area of the region of interest [27]. As a result, a subset of these important voxels with obvious velocity information was most likely included without any correction. By contrast, the flow rate measurement in this study was higher than that reported by Pierre Bouillot et al. [27], although the pre-processing approach was used in both. This difference can be explained by, in the present study, the subjects were much younger (35 vs. 53 years old on average); ageing could also affect the flow rate. In a study using 4D flow MRI for the assessment of cerebral blood flow, Wu et al. [32] identified that the cerebral blood flow parameters are highly associated with age. Another study by Zhao et al. [33] confirmed that the cerebral arterial blood flow decreases significantly with age.

In the 3D blood flow pattern visualized by CFD and 4D flow MRI in this study, the results identified several differences as the primary limitation in using 4D flow MRI is the spatio-temporal resolution. The limited spatial and temporal resolution leads to an insufficient number of velocity data points within the fluid flow from which to estimate model parameters [18]. As a result, 4D flow MRI visualization displays poorer complex flow patterns quality compared with CFD models, i.e., more parallel flows, less swirling, and fewer transition flows in the ICA bifurcation.

The velocity fields from the CFD and 4D flow MRI showed large differences at the ACA A2. This may be due to deviation in the geometry created by the two approaches. The majority of these issues stem from the difficulty of image segmentation as a result of poor image contrast of 4D flow MRI. The unprecise geometric segmentation in the distal ACA created by the 4D flow MRI may introduce an error in velocity magnitude estimation. Moreover, the influence of inadequate resolution can also be noticed from the shape of 4D flow MRI profiles. This impact is predominantly present in the small vessels, where the vessels are tortuous and therefore cannot be covered by as many voxels [9,17,34].

To overcome these constraints and to recreate denoised, divergence-free, high-resolution flow fields, the current approach combined 4D flow MRI and CFD. The systematic use of 4D flow MRI measurements and the methodology of adapted flow rate extraction provided subject-specific inflow rates required for reliable simulation.

The CFD overcame the limitations of 4D flow MRI in image segmentation, rendering them with a much finer spatial resolution. By applying complementary vessel geometry information from TOF-MRA with higher spatial resolution and contrast than 4D flow MRI, the CFD had advantages in demonstrating the 3D vessel geometry and outlining the arterial wall. Since TOF-MRA is a mandatory neurovascular disease examination, no additional acquisition time was required.

As can be seen in the 3D blood flow pattern derived by the two methods, the CFD reconstructed result was not only able to recover the underlying blood flow characteristics, but also greatly improved streamline lengths. The blood flow through the ACoA and the low flows near the vessel wall were more accurately captured by the CFD than the 4D flow MRI. This demonstrates that the current algorithm can recreate high-resolution velocity profiles from noisy 4D flow MRI data with low resolution. Moreover, recirculation regions at the bifurcation visualized by CFD are of higher quality compared with 4D flow MRI. Visualization of recirculation regions is essential for clinicians when evaluating vascular disease [35]. Therefore, this complementary method could better provide insight into the pathophysiology of cerebrovascular disease.

We used both CFD and TOF-MRA SIG as methods for representing WSS. Although a relative agreement was observed between TOF-MRA SIG and CFD WSS, the discrepancy between the two methods remained obvious in the visualization of small vessels (i.e., ACA and ACoA) and at the ICA bifurcation. These differences resulted from the limitations of the TOF-MRA SIG methods, which displayed the relative difference in signal intensity along the arterial wall. As the imaging signal is visualized poorer at small arteries, the resolutions of MRI are inadequate to accurately resolve the signal intensity gradient. The TOF-MRA sequence has proved sensitive to signal loss induced by flow disturbances [36,37], which may limit its application at the locations of the vascular systems where the vessels are relatively complex, such as the ICA bifurcation.

Despite these limitations, the TOF-MRA SIG method has the potential to aid in clinical management. With a workflow that takes less than 5 min, the present study suggests that TOF-MRA SIG could provide specific hemodynamic information for each subject. Thus, it can help clinicians make rapid decisions by offering an effective screening process regarding the risk of cardiovascular disease for a patient and in deciding whether or not further examination is required [15].

Despite CFD being currently the best approach for WSS analysis, its limitations have been noted [38,39]. Wey assumed that the vessel walls were rigid, reducing CFD processing complexity. However, in reality, the arterial wall is not rigid and is known to have inherent elasticity, which reduces the WSS [40]. Therefore, the discrepancy of WSS estimations derived by the two methods may not just be due to the lower resolution of TOF-MRA SIG, but may also be related to a relative overestimation of the CFD simulation.

The current study had several limitations. We did not conduct a comparison between the 4D flow MRI and CFD for velocity measurement, as the use of 4D flow MRI data as inlet boundary conditions for CFD simulations could introduce bias for subsequent comparison data of the two methods. However, the purpose of this study was merely to test the capability of the two techniques to generate a 3D specific model. The present methods improve the clinical application feasibility with subject-specific models.

A further limitation was the spatio-temporal resolution used in the MRI scans. With a scan time of 20 min in the conventional 3D PC MRI acquisition, spatial resolution could not be better than 1.1 mm^3^. A longer PC MRI scan time would enable these resolutions to improve, but would also increase the likelihood of artifacts due to patient motion, which can result in a substantial reduction in the quality of MR images. Recent developments in the technique for accelerating PC MRI acquisition will lead to the widespread implementation of 4D flow MRI in clinical trials [41].

For CFD, the limitations related to the CFD models were due to the simplifications of the current approach. In this study, blood was considered as Newtonian viscosity fluid. However, blood actually behaves as a non-Newtonian fluid of which viscosity continuously changes according to shear rates. If this non-Newtonian viscosity change was applied as a boundary condition, a large variation in wall shear stress would be observed in the ICA bifurcations due to sudden changes in shear rates. The setup of boundary conditions is of great importance for solving the Navier–Stokes equations, but the validity of the outlet boundary conditions application is still debatable [38,39]. In the present study, the traction-free outlet boundary condition was applied. This approach was chosen due to the lack of data regarding absolute pressure in the various outlet branches. Since current imaging techniques cannot realistically visualize very small intracranial arteries, these small branches are overlooked in such computational studies. Thus, the calculated sum of all outlet blood flow rates results in a smaller value compared to total inlet flow rates in CFD simulations [28].

We also did not perform a comparison between the 4D flow MRI and TOF-MRA SIG for determining WSS. 4D flow MRI, which is based on dynamic phase display of the flow, can provide the arterial WSS distribution [7], but previous studies have reported that it is not appropriate for analyzing arterial WSS [29,39]. In clinical practice, the use of 4D flow MRI for estimating WSS remains limited due to the unclear relevance of WSS and concerns about its accuracy due to poor spatial resolution along the arterial wall [15].

Finally, while the methods of 4D flow MRI, TOF-MRA SIG, and CFD were successfully performed in healthy subjects, further studies are required to decide whether this multimodality approach is also accurate for potential applications in elderly subjects and patients with neurovascular disease.

## 5. Conclusions

In this study, a multimodality analysis was conducted using 4D flow MRI, TOF-MRA SIG, and CFD to investigate the 3D blood flow characteristics and WSS of the cerebral arteries. Consistent with previous references, we identified that 4D flow MRI allows a direct and accurate quantification of the velocity magnitudes of the major cerebral arteries. Our approach combines 4D flow MRI data and subject-specific CFD simulation and can recreate the underlying 3D blood flow pattern at higher spatial resolutions. This current study also showed that TOF-MRA SIG and CFD WSS of the major cerebral arteries were reasonably in agreement with the locations where the arterial WSS was relatively high. However, further studies are required for the validation of this new method of SIG in the assessment of WSS in cerebral arteries, especially for small vessels and bifurcations.

## Figures and Tables

**Table 1:** Comparison of previously reported data of mean flow rates calculated with PC MRI in healthy volunteers.

Study	Vascular Segment			PC MRI	Correction Schemes
	ICA	M1	A1		
Wahlin [24]	4.0 (0.6)	2.3 (0.3)	1.4 (0.5)	2D	No correction
Zarrinkoob [25]	4.3 (0.8)	2.4 (0.5)	1.4 (0.3)	2D	No correction
MacDonald [26]	4.9 (1.5)	2.4 (0.8)	1.7 (0.7)	3D	No correction
Bouillot [27]	3.4 (0.7)	1.9 (0.5)	1.1 (0.4)	3D	Correction
This study	3.8 (0.8)	2.5 (0.4)	1.2 (0.5)	3D	Correction

Mean flow rates in mL/s, standard deviation in parenthesis. ICA: internal carotid arter-ies-paraophthalmic segment, M1: middle cerebral arteries-middle M1 segment, A1: anterior cerebral arteries-middle A1 segment, PC MRI: phase-contrast magnetic resonance imaging.

## Data Availability

The data presented in this study are available on request from the corresponding author. The data are not publicly available due to privacy restrictions.

## References

[1] Donkor E.S. (2018). Stroke in the 21st Century: A Snapshot of the Burden, Epidemiology, and Quality of Life. Stroke Res. Treat..

[2] Absher J.R., Madeline L., Webb S.W., Rayes M. (2018). Cerebrovascular Disease. Reference Module in Neuroscience and Biobehavioral Psychology.

[3] Prado C.M., Ramos S.G., Alves-Filho J.C., Elias J., Cunha F.Q., Rossi A.M. (2006). Turbulent flow/low wall shear stress and stretch differentially affect aorta remodeling in rats. J. Hypertens..

[4] MacMahon S., Peto R., Collins R., Godwin J., Cutler J., Sorlie P., Abbott R., Neaton J., Dyer A., Stamler J. (1990). Blood pressure, stroke, and coronary heart disease *1Part 1, prolonged differences in blood pressure: Prospective observational studies corrected for the regression dilution bias. Lancet.

[5] Mendez V., Di Giuseppe M., Pasta S. (2018). Comparison of hemodynamic and structural indices of ascending thoracic aortic aneurysm as predicted by 2-way FSI, CFD rigid wall simulation and patient-specific displacement-based FEA. Comput. Biol. Med..

[6] Thim T., Hagensen M.K., Falk E., Hørlyck A., Kim W.Y., Niemann A.K., Thrysøe S.A., Drouet L., Paaske W.P., Bøtker H.E. (2012). Wall shear stress and local plaque development in stenosed carotid arteries of hypercholesterolemic minipigs. J. Cardiovasc. Dis. Res..

[7] Van Ooij P., Potters W.V., Guédon A., Schneiders J.J., Marquering H.A., Majoie C.B., Van Bavel E., Nederveen A.J. (2013). Wall shear stress estimated with phase contrast MRI in an in vitro and in vivo intracranial aneurysm. J. Magn. Reson. Imaging.

[8] Rinaudo A., Raffa G.M., Scardulla F., Pilato M., Scardulla C., Pasta S. (2015). Biomechanical implications of excessive endograft protrusion into the aortic arch after thoracic endovascular repair. Comput. Biol. Med..

[9] Schnell S., Wu C., Ansari S.A. (2016). Four-dimensional MRI flow examinations in cerebral and extracerebral vessels—ready for clinical routine?. Curr. Opin. Neurol..

[10] Chien A., Tateshima S., Sayre J., Castro M., Cebral J., Viñuela F. (2009). Patient-specific hemodynamic analysis of small internal carotid artery-ophthalmic artery aneurysms. Surg. Neurol..

[11] Shojima M., Oshima M., Takagi K., Torii R., Hayakawa M., Katada K., Morita A., Kirino T. (2004). Magnitude and Role of Wall Shear Stress on Cerebral Aneurysm. Stroke.

[12] Hoi Y., Woodward S.H., Kim M., Taulbee D.B., Meng H. (2006). Validation of CFD Simulations of Cerebral Aneurysms with Implication of Geometric Variations. J. Biomech. Eng..

[13] Nishino K., Kawaguchi D., Sato H., Isoda H., Kosugi T. (2004). In vitro PIV measurement and CFD analysis of flow patterns in cerebral aneurysm. J. Vis. Soc. Jpn..

[14] Ford M.D., Nikolov H.N., Milner J.S., Lownie S.P., Demont E.M., Kalata W., Loth F., Holdsworth D.W., Steinman D.A. (2008). PIV-Measured Versus CFD-Predicted Flow Dynamics in Anatomically Realistic Cerebral Aneurysm Models. J. Biomech. Eng..

[15] Han K.-S., Lee S.H., Ryu H.U., Park S.-H., Chung G.-H., Cho Y.I., Jeong S.-K. (2017). Direct Assessment of Wall Shear Stress by Signal Intensity Gradient from Time-of-Flight Magnetic Resonance Angiography. BioMed Res. Int..

[16] Dyverfeldt P., Bissell M., Barker A.J., Bolger A.F., Carlhäll C.-J., Ebbers T., Francios C.J., Frydrychowicz A., Geiger J., Giese D. (2015). 4D flow cardiovascular magnetic resonance consensus statement. J. Cardiovasc. Magn. Reson..

[17] Ha H., Kim G.B., Kweon J., Lee S.J., Kim Y.-H., Lee D.H., Yang D.H., Kim N. (2016). Hemodynamic Measurement Using Four-Dimensional Phase-Contrast MRI: Quantification of Hemodynamic Parameters and Clinical Applications. Korean J. Radiol..

[18] Cebral J.R., Putman C.M., Alley M.T., Hope T., Bammer R., Calamante F. (2009). Hemodynamics in normal cerebral arteries: Qualitative comparison of 4D phase-contrast magnetic resonance and image-based computational fluid dynamics. J. Eng. Math..

[19] Sun W., Ruan Z., Dai X., Li S., Li S., Zhang J., Chen J., Zhang H., Xu H. (2018). Quantifying Hemodynamic Changes in Moyamoya Disease Based on Two-Dimensional Cine Phase-Contrast Magnetic Resonance Imaging and Computational Fluid Dynamics. World Neurosurg..

[20] Cebral J.R., Castro M.A., Soto O., Löhner R., Alperin N. (2003). Blood-flow models of the circle of Willis from magnetic resonance data. J. Eng. Math..

[21] Isoda H., Ohkura Y., Kosugi T., Hirano M., Alley M.T., Bammer R., Pelc N.J., Namba H., Sakahara H. (2009). Comparison of hemodynamics of intracranial aneurysms between MR fluid dynamics using 3D cine phase-contrast MRI and MR-based computational fluid dynamics. Neuroradiology.

[22] Fukazawa K., Ishida F., Umeda Y., Miura Y., Shimosaka S., Matsushima S., Taki W., Suzuki H. (2015). Using Computational Fluid Dynamics Analysis to Characterize Local Hemodynamic Features of Middle Cerebral Artery Aneurysm Rupture Points. World Neurosurg..

[23] Lee W.-J., Jeong S.-K., Han K.-S., Lee S.H., Ryu Y.J., Sohn C.-H., Jung K.-H. (2020). Impact of Endothelial Shear Stress on the Bilateral Progression of Unilateral Moyamoya Disease. Stroke.

[24] Wahlin A., Ambarki K., Birgander R., Wieben O., Johnson K., Malm J., Eklund A. (2013). Measuring Pulsatile Flow in Cerebral Arteries Using 4D Phase-Contrast MR Imaging. Am. J. Neuroradiol..

[25] Zarrinkoob L., Ambarki K., Wåhlin A., Birgander R., Eklund A., Malm J. (2015). Blood Flow Distribution in Cerebral Arteries. Br. J. Pharmacol..

[26] Macdonald M.E., Frayne R. (2015). Phase contrast MR imaging measurements of blood flow in healthy human cerebral vessel segments. Physiol. Meas..

[27] Bouillot P., Delattre B.M.A., Brina O., Ouared R., Farhat M., Chnafa C., Steinman D.A., Lovblad K., Pereira V.M., Vargas M.I. (2018). 3D phase contrast MRI: Partial volume correction for robust blood flow quantification in small intracranial vessels. Magn. Reson. Med..

[28] Berg P., Stucht D., Janiga G., Beuing O., Speck O., Thévenin D. (2014). Cerebral Blood Flow in a Healthy Circle of Willis and Two Intracranial Aneurysms: Computational Fluid Dynamics Versus Four-Dimensional Phase-Contrast Magnetic Resonance Imaging. J. Biomech. Eng..

[29] Boussel L., Rayz V., Martin A., Acevedo-Bolton G., Lawton M.T., Higashida R., Smith W.S., Young W.L., Saloner D. (2009). Phase-contrast magnetic resonance imaging measurements in intracranial aneurysms in vivo of flow patterns, velocity fields, and wall shear stress: Comparison with computational fluid dynamics. Magn. Reson. Med..

[30] Hollnagel D.I., Summers P.E., Poulikakosb D., Kolliasa S.S. (2009). Comparative velocity investigations in cerebral arteries and aneurysms: 3D phase-contrast MR angiography, laser Doppler velocimetry and computational fluid dynamics. NMR Biomed..

[31] Ngo M.T., Kim C.I., Jung J., Chung G.H., Lee D.H., Kwak H.S. (2019). Four-Dimensional Flow Magnetic Resonance Imaging for Assessment of Velocity Magnitudes and Flow Patterns in The Human Carotid Artery Bifurcation: Comparison with Computational Fluid Dynamics. Diagnostic.

[32] Wu C., Honarmand A.R., Schnell S., Kuhn R., Schoeneman S.E., Ansari S.A., Carr J., Markl M., Shaibani A. (2016). Age-Related Changes of Normal Cerebral and Cardiac Blood Flow in Children and Adults Aged 7 Months to 61 Years. J. Am. Hear. Assoc..

[33] Zhao X., Zhao M., Du X., Ruland S., Charbel F.T., Amin-Hanjani S. (2014). Wall Shear Stress in Major Cerebral Arteries as a Function of Age and Gender-A Study of 301 Healthy Volunteers. J. Neuroimaging.

[34] Harloff A., Albrecht F., Spreer J., Stalder A.F., Bock J., Frydrychowicz A., Schöllhorn J., Hetzel A., Schumacher M., Hennig J. (2008). 3D blood flow characteristics in the carotid artery bifurcation assessed by flow-sensitive 4D MRI at 3T. Magn. Reson. Med..

[35] Ku D.N. (1997). Blood Flow in Arteries. Annu. Rev. Fluid Mech..

[36] Chen Z., Qin H., Liu J., Wu B., Cheng Z., Jiang Y., Liu L., Jing L., Leng X., Jing J. (2020). Characteristics of Wall Shear Stress and Pressure of Intracranial Atherosclerosis Analyzed by a Computational Fluid Dynamics Model: A Pilot Study. Front. Neurol..

[37] Urchuk S.N., Plewes D.B. (1992). Mechanisms of flow-induced signal loss in MR angiography. J. Magn. Reson. Imaging.

[38] Cibis M., Potters W.V., Gijsen F.J.H., Marquering H., VanBavel E., Van Der Steen A.F.W., Nederveen A.J., Wentzel J.J. (2014). Wall shear stress calculations based on 3D cine phase contrast MRI and computational fluid dynamics: A comparison study in healthy carotid arteries. NMR Biomed..

[39] Szajer J., Ho-Shon K. (2018). A comparison of 4D flow MRI-derived wall shear stress with computational fluid dynamics methods for intracranial aneurysms and carotid bifurcations—A review. Magn. Reson. Imaging.

[40] Lantz J., Renner J., Karlsson M. (2011). Wall Shear Stress in a Subject Specific Human Aorta—Influence of Fluid-Structure Interaction. Int. J. Appl. Mech..

[41] Dyvorne H., Knight-Greenfield A., Jajamovich G., Besa C., Cui Y., Stalder A., Markl M., Taouli B. (2015). Abdominal 4D Flow MR Imaging in a Breath Hold: Combination of Spiral Sampling and Dynamic Compressed Sensing for Highly Accelerated Acquisition. Radiology.

